# 

**Published:** 1890-09

**Authors:** 


### CONTENTS—SEPTEMBER, 1890.—VOL. VI., NO. 3.

ORIGINAL ARTICLES— PAGE.

Cystic Tumors of the Inferior Maxilla. By Prof. W. B. Rogers, Memphis .......... 97

Laparotomy for Fibro-Cystoma: The Case of Natividad Krishbaum. By Dr. D. Berrey, San Antonio .......... 107

Gunshot Wound of the Abdomen. A case by Sam Cummingham, M. D., Elgin .......... 110

Removal by Tracheotomy of a Safety Pin from Larynx After One Year. By Dr. H. L. Fountain, Bryan .......... 112

CORRESPONDENCE—

Letters from Europe: The Gieat Hospitals: The Congress, etc. By Dr. E. J. Beall, Fort Worth .......... 113

SOCIETY NOTES—

Austin District Medical Society .......... 116

Travis County Medical Society. North West Texas Medical Association, etc .......... 117

EDITORIAL—

Quacks in and Out of the Profession .......... 119

A Plan Suggested .......... 123

Was it a Consideration for theWelfare of the Inmates,—or Politics? .......... 125

The "Cosmopolitan," Club Rates .......... 127

MEDICAL NEWS AND MISCELLANY—

Many Items .......... 118, 127, 128

BOOK NOTICES .......... 131

PUBLISHER'S NOTES .......... 136

